# Transcatheter Interventions in Infants With Congenital Heart Disease: A Two-Year Retrospective Case Series From a Tertiary Centre in India

**DOI:** 10.7759/cureus.105948

**Published:** 2026-03-26

**Authors:** Ramesh Patel, Gaurav K Mittal, Jai Bharat Sharma

**Affiliations:** 1 Cardiology, Geetanjali Medical College and Hospital, Udaipur, IND

**Keywords:** congenital heart disease, infants, interventional cardiology, neonatal mortality, procedural success, transcatheter interventions

## Abstract

Background: Severe forms of congenital heart disease (CHD) remain a leading cause of neonatal morbidity and mortality. In recent decades, transcatheter interventions have emerged as a less invasive, repeatable, and effective alternative to surgical treatment, particularly for critically ill neonates.

Objective: This retrospective study evaluates the demographic characteristics, diagnoses, procedures, complications, and 30-day outcomes of infants undergoing transcatheter interventions for CHD at Geetanjali Medical College and Hospital, Udaipur, Rajasthan, between December 2021 and November 2023.

Methods: Of 108 infants admitted with CHD during the study period, 28 underwent transcatheter interventions. Patients managed surgically or medically, or whose parents declined intervention, were excluded.

Results: Transcatheter interventions were successfully performed in 28 infants with CHD over two years (2021-2023). Acyanotic CHD (n = 9) included seven patent ductus arteriosus (PDA) and two ventricular septal defect (VSD) device closures (median age = 11 months, median weight = 6 kg) with 100% procedural success and 30-day survival. Patients with obstructive CHD (n = 5) underwent balloon pulmonary valvotomy and coarctoplasty (median age = 5 months, median weight = 5.6 kg), achieving universal success and 100% 30-day survival. Patients with cyanotic CHD (n = 14) received balloon atrial septostomy (n = 5), right ventricular outflow tract stenting (n = 2), and PDA stenting (n = 7) with 93% success; one PDA stenting failure occurred alongside two cases of arrhythmias, with 50% 30-day survival cumulatively.

Conclusion: Transcatheter interventions are a potentially valuable modality for safe and effective definitive or bridge to definitive treatment of infants with CHD, especially in resource-limited settings. These procedures may play a vital role in bridging the gap in paediatric cardiac care.

## Introduction

Severe congenital heart disease (CHD) is a significant contributor to neonatal morbidity and mortality, affecting approximately 2.5-3 per 1,000 live births [1]. Globally, a 2024 meta-analysis reported an increasing annual prevalence of CHD in infants, with ventricular and atrial septal defects contributing the highest burden [2]. A landmark 2025 global epidemiological analysis found over 4.18 million children under five years living with CHD across 204 countries in 2021, with India bearing the single largest national count at an estimated 725,281 cases [3]. In India, approximately 200,000-250,000 babies are born with CHD annually, of whom nearly one-third have critical lesions that are often fatal without timely intervention [4]. Despite this enormous burden, advanced cardiac care is accessible to only a minority [5]. Approximately 300 paediatric cardiologists practise in a handful of tertiary centres, predominantly in urban and metropolitan regions, with major states such as Uttar Pradesh and Bihar, accounting for tens of millions of annual births, lacking even a single centre capable of performing neonatal cardiac surgery [6]. These critical lesions often strike in the earliest days of life, creating urgent threats through obstructed blood flow, improper shunting, or inadequate mixing that starve vital organs of oxygen and perfusion. While surgery has long been the cornerstone of definitive repair, the rise of transcatheter interventions has offered a lifeline, especially for fragile infants, where the scalpel's risks might outweigh the rewards. The pioneering balloon atrial septostomy (BAS) procedure by Rashkind in the 1960s marked the advent of interventional cardiology in neonates [7]. Since then, through decades of ingenuity, remarkable advancements have transformed transcatheter techniques into vital therapeutic options, particularly for critically ill infants, offering lower morbidity, greater repeatability, and improved outcomes compared to surgery [8].

Yet, in low- and middle-income countries such as India, where surgical waitlists can stretch, and neonatal transport poses its own perils, real-world data on infant outcomes remain all too scarce. Against this backdrop, our retrospective study conducted at Geetanjali Medical College and Hospital reviewed the case records, intervention types, complications encountered, and 30-day outcome of 28 infants treated for acyanotic, obstructive, and cyanotic CHD via catheter from 2021 to 2023. By focusing exclusively on infants, the most time-sensitive and highest-risk subgroup, and reporting from a resource-constrained real-world setting, this study addresses a critical evidence gap in paediatric interventional cardiology in India.

## Materials and methods

Ethics approval

This retrospective case series was approved by the Institutional Ethics Committee of Geetanjali Medical College and Hospital, Udaipur, Rajasthan (Approval No. 96/2025). A waiver of informed consent was granted on the basis of the retrospective, non-interventional study design. The study was conducted in accordance with the Declaration of Helsinki (revised 2013).

Study design and setting

This was a single-centre retrospective case series conducted at Geetanjali Medical College and Hospital, Udaipur, Rajasthan, a tertiary academic referral centre serving a large semi-urban and rural catchment population in southern Rajasthan. The centre houses a dedicated cardiac catheterisation laboratory with paediatric cardiac intervention capability and provides cardiology and paediatric cardiology services to patients referred from across the region.

Study period

Medical records were reviewed for all eligible patients admitted between December 2021 and November 2023 (24-month period). This study period was selected to reflect the initial two-year institutional experience with transcatheter interventions in infants following the establishment of a dedicated paediatric cardiac catheterisation programme.

Study population

Infants were defined as children aged ≤12 months at the time of the transcatheter procedure, consistent with standard WHO age classification. This age group was specifically targeted because infants and neonates, in particular, represent the most haemodynamically vulnerable subset of the CHD population, with the highest procedure-related risk, the greatest time-sensitivity, and the most significant evidence gap in Indian literature.

Inclusion criteria included the following: age ≤12 months at the time of the transcatheter intervention; confirmed diagnosis of CHD by two-dimensional echocardiography with Doppler assessment performed by an experienced cardiologist; cardiac CT angiography or diagnostic catheterisation was additionally used in selected cases requiring further anatomical delineation; transcatheter intervention performed as the primary treatment strategy during the study period; and complete procedural records and 30-day outcome data available.

Exclusion criteria included the following: conservative (medical) management as the definitive treatment strategy; primary surgical intervention without a preceding transcatheter attempt; guardian's refusal of transcatheter procedures; and incomplete procedural or 30-day follow-up records.

A total of 108 infants were admitted with CHD during the study period. Of these, 28 met the inclusion criteria and underwent transcatheter intervention; the remaining 80 were managed conservatively, referred for surgery, or declined intervention and were excluded.

Procedural details

All transcatheter interventions were performed in a dedicated cardiac catheterisation laboratory under general anaesthesia or deep sedation, with continuous haemodynamic monitoring, fluoroscopic guidance, and real-time echocardiographic support. Vascular access was obtained via the femoral vein or femoral artery using the standard Seldinger technique; umbilical venous access was utilised in neonates where feasible and anatomically appropriate.

Balloon atrial septostomy (BAS) was performed using a Rashkind balloon catheter (Edwards Lifesciences, Irvine, CA, USA) or a static Z-Med balloon catheter (NuMED Inc., Hopkinton, NY, USA), with septostomy size guided by real-time echocardiography.

Balloon pulmonary valvuloplasty (BPV) and balloon aortic valvuloplasty (BAV) were performed using appropriately sized Tyshak or Z-Med balloon catheters (NuMED Inc.); balloon sizes were selected to achieve a balloon-to-annulus ratio of 1.0-1.2 for pulmonary and 0.85-1.0 for aortic valvuloplasty.

Ductal stenting and right ventricular outflow tract (RVOT) stenting, when performed, used premounted coronary stents sized according to ductal or vessel diameter on angiography.

All procedures were performed by or under the direct supervision of an interventional cardiologist with dedicated paediatric catheterisation experience consistent with institutional standards.

Outcome definitions

The primary outcome was procedural success, and procedures were defined specifically as follows.

Procedural success for BAS was defined as the creation of an unrestrictive interatrial communication ≥6 mm in diameter on post-procedural echocardiography, accompanied by an improvement in arterial oxygen saturation of ≥10% from pre-procedural baseline.

Success for BPV was defined as post-procedural reduction of peak instantaneous Doppler gradient by ≥50% from baseline, or residual peak gradient <35 mmHg.

Procedural success for BAV was defined as post-procedural peak-to-peak gradient reduction ≥50%, without the development of more than mild aortic regurgitation.

Success for ductal/pulmonary artery stenting was defined as angiographic confirmation of stent patency with restoration of antegrade pulmonary blood flow and clinical improvement in oxygen saturation.

Secondary outcomes were 30-day all-cause mortality.

Procedural complications were defined as any of the following: cardiac perforation or tamponade, vessel injury requiring intervention, clinically significant arrhythmia requiring treatment, new or worsened aortic regurgitation (≥moderate) following BAV, device embolisation, valvular or subvalvular trauma, access-site haematoma requiring blood transfusion, or unplanned repeat intervention or escalation to surgery within 30 days of the index procedure.

Data collection

Demographic, clinical, procedural, and outcome variables were retrieved from electronic medical records and paper case files by two independent investigators. Data collected included age, sex, weight, presenting diagnosis, type of CHD, clinical acuity at presentation, echocardiographic parameters, procedural details, device specifications, immediate post-procedural haemodynamics, complications, and 30-day outcomes.

Missing data were not imputed. Variables with missing values are reported alongside their respective available denominators. Cases with missing 30-day outcome data were excluded from mortality and complication analyses and are documented in the study flow diagram.

Statistical analysis

All statistical analyses were performed using SPSS version 25.0 (IBM Corp., Armonk, NY, USA). Categorical variables are expressed as frequency and percentage (n, %). Continuous variables are expressed as mean ± standard deviation (SD) for normally distributed data and as median with interquartile range (IQR) for non-normally distributed data; normality was assessed using the Shapiro-Wilk test.

Subgroup comparisons across intervention categories (BAS, balloon valvuloplasty, ductal/pulmonary artery stenting) were performed using Fisher's exact test for categorical variables and the Kruskal-Wallis test for continuous variables, given the small sample sizes in each subgroup. A two-tailed p-value of <0.05 was considered statistically significant.

Given the retrospective, descriptive, single-centre nature of this case series and the small sample size (n = 28), all statistical comparisons are exploratory and hypothesis-generating only. No formal a priori hypothesis testing was pre-specified, and no adjustment for multiple comparisons was applied. The findings should be interpreted within these methodological limitations.

## Results

In the acyanotic CHD group, nine infants underwent device closure, including seven with patent ductus arteriosus (PDA) and two with ventricular septal defect (VSD) (Table 1). These procedures were performed at a median age of approximately 11 months (IQR: 330-344 days) and a median weight close to 6 kg, exclusively via femoral access, and achieved 100% procedural success and 100% 30‑day survival. A single intraprocedural episode of paroxysmal supraventricular tachycardia occurred in one older infant undergoing PDA closure, which reverted spontaneously without pharmacological therapy, yielding an overall complication rate of 11.1% in this subgroup and no need for re‑intervention, intensive care escalation, or prolonged hospitalisation.

**Table 1:** Profile of infants with transcatheter intervention in acyanotic congenital heart disease. PDA: patent ductus arteriosus; VSD: ventricular septal defect; PSVT: paroxysmal supraventricular tachycardia.

Age	Gender	Weight in kg	Diagnosis	Procedure	Access	Complication	30-day survival
11 months and 20 days	F	6	PDA	Device closure	Femoral	Nil	Yes
11 months and 9 days	F	5	PDA	Device closure	Femoral	Nil	Yes
11 months and 2 days	F	4.3	PDA	Device closure	Femoral	Nil	Yes
9 months and 7 days	M	6	PDA	Device closure	Femoral	Nil	Yes
11 months	F	6	PDA	Device closure	Femoral	Nil	Yes
10 months and 29 days	M	4.1	PDA	Device closure	Femoral	Nil	Yes
11 months and 14 days	M	8.35	PDA	Device closure	Femoral	PSVT	Yes
10 months and 26 days	F	7	VSD	Device closure	Femoral	Nil	Yes
10 months and 2 days	F	6	VSD	Device closure	Femoral	Nil	Yes

Among infants with obstructive CHD, two underwent BPV, and three underwent balloon coarctoplasty, with one of the latter also receiving concomitant BAV for severe aortic stenosis (Table 2). This subgroup, which was intervened at a median age of around five months (IQR: 81-155 days) and with weights between 3.3 and 6.2 kg (median = 5.6 kg), also demonstrated 100% procedural success and 100% 30‑day survival. One child developed an acute femoral deep vein thrombosis on day two after BPV, which was managed successfully with low‑molecular‑weight heparin without long‑term sequelae, corresponding to a 20% complication rate in the obstructive cohort and no requirement for further procedures or ICU escalation.

**Table 2:** Profile of infants with transcatheter intervention in obstructive heart disease. AS: aortic stenosis; CoA: coarctation of the aorta; PS: pulmonary stenosis; BPV: balloon pulmonary valvuloplasty; BAV: balloon aortic valvuloplasty; DVT: deep vein thrombosis.

Age	Gender	Weight in kg	Diagnosis	Procedure	Access	Complication	30-day survival
5 months and 3 days	M	5.7	Severe valvular PS	BPV	Femoral	Nil	Yes
5 months and 1 day	M	5.6	Severe valvular PS	BPV	Femoral	Acute DVT	Yes
8 months and 1 day	M	6.2	CoA, moderate eccentric mitral regurgitation	Coarctoplasty	Femoral	Nil	Yes
2 months and 20 days	M	3.3	CoA	Coarctoplasty	Femoral	Nil	Yes
2 months and 14 days	F	4.2	Severe AS with CoA	BAV and coarctoplasty	Femoral	Nil	Yes

In the cyanotic CHD group, 14 infants underwent palliative transcatheter procedures, with five infants undergoing BAS, two RVOT stents, and seven PDA stents (Table 3). BAS was performed predominantly in neonates with dextro-transposition of the great arteries (d‑TGA) at a median age of two days and median weight of 2.7 kg, using umbilical or femoral venous access, and achieved 100% technical success but only 40% 30‑day survival. One BAS patient developed atrial fibrillation that reverted with medical therapy, and all three deaths occurred in very low‑weight, haemodynamically unstable neonates with d‑TGA in whom the septostomy was technically adequate, but definitive arterial switch surgery could not be performed within 30 days.

**Table 3:** Profile of infants with transcatheter intervention in cyanotic congenital heart disease. ASD: atrial septal defect; FV: femoral vein; dTGA: dextro-transposition of the great arteries; PS: pulmonary stenosis; PA: pulmonary atresia; RV: right ventricle; TOF: tetralogy of Fallot; UV: umbilical vein; VT: ventricular tachycardia; VSD: ventricular septal defect; DILV: double inlet left ventricle; BAS: balloon atrial septostomy; RVOT: right ventricular outflow tract; PDA: patent ductus arteriosus; AF: atrial fibrillation.

Age	Gender	Weight in kg	Diagnosis	Procedure	Access	Complication	30-day survival
4 months and 9 days	M	4.1	Tricuspid atresia Ib with restrictive ASD	BAS	FV	Nil	Yes
2 days	M	2.4	dTGA	BAS	UV	AF	Yes
1 day	F	2.7	dTGA	BAS	UV	Nil	No
1 day	M	2.8	dTGA	BAS	UV	Nil	No
9 days	M	2.5	dTGA	BAS	FV	Nil	No
10 months and 23 days	F	5	TOF	RVOT stenting	FV	Nil	Yes
9 months	M	4	TOF	RVOT stenting	FV	Nil	Yes
4 months and 1 day	F	2.7	TOF pulmonary atresia	PDA stenting	Femoral	Nil	No
3 days	M	2	Hypoplastic RV with severe PS	PDA stenting	Femoral	Nil	No
2 months and 20 days	F	3.5	Tricuspid atresia/restrictive VSD/large ASD	PDA stenting	Femoral	VT	Yes
4 months and 2 days	M	5.1	TOF with PA	PDA stenting	Femoral	Failed	No
10 days	M	2.4	Dextrocardia with DILV, hypoplastic RV, large VSD, severe PS	PDA stenting	Femoral	Nil	No
3 days	F	3.1	TOF with PA	PDA stenting	Left axillary	Second attempt	Yes
1 day	M	3.3	TOF with PA	PDA stenting	Femoral	Nil	Yes

RVOT stenting was undertaken in two older infants with tetralogy of Fallot (TOF), both via femoral venous access, and was uniformly successful, with no complications and 100% 30‑day survival. PDA stenting was performed in seven neonates and young infants with complex duct‑dependent or single‑ventricle anatomies, at a median age of 10 days and median weight of 3.1 kg; technical success was achieved in six of seven cases (86%), and 30‑day survival in this subgroup was 43%. Complications occurred in three PDA stent cases (43%): one episode of ventricular tachycardia requiring successful cardioversion, one procedural failure due to tortuous ductal anatomy, and one case necessitating a second stent attempt via the left axillary artery. Four infants in the PDA stent group died, all of whom were neonates or young infants with complex duct‑dependent or single‑ventricle lesions and low body weight; three deaths followed technically successful stenting and were attributed to underlying disease severity and intercurrent illness, while one occurred in the infant with failed stent placement.

Procedural success rate

The overall transcatheter procedural success rate was 96% (27/28). The single procedural failure occurred in the cyanotic subgroup, in a four-month-old male infant with tetralogy of Fallot and pulmonary atresia (TOF-PA), in whom PDA stenting could not be completed due to tortuous ductal anatomy precluding a stable wire position; this infant did not survive to 30 days. A separate TOF-PA case in this subgroup required a second procedural attempt via left axillary access after the initial femoral approach failed. This was ultimately technically successful, and the infant survived. This case is recorded as a successful procedure with a vascular access complication, not as a procedural failure.

Mortality analysis

Seven deaths occurred within the 30-day follow-up period, all in the cyanotic subgroup (three BAS, four PDA stenting) (Table 4). No deaths occurred in the acyanotic or obstructive groups. None of the seven deaths was directly attributable to a procedural complication. Six of seven patients died despite technically successful procedures, reflecting the natural history of complex duct-dependent cyanotic lesions in resource-constrained settings where staged surgical palliation was not immediately available.

**Table 4:** Comparison of non‑cyanotic (acyanotic + obstructive) vs. cyanotic congenital heart disease infants undergoing transcatheter intervention. Non‑cyanotic CHD includes acyanotic (PDA/VSD device closure) and obstructive (BPV, coarctoplasty, BAV) lesions. CHD: congenital heart disease; PDA: patent ductus arteriosus; VSD: ventricular septal defect; BPV: balloon pulmonary valvuloplasty; BAV: balloon aortic valvuloplasty.

Variable	Non‑cyanotic CHD (n = 14)	Cyanotic CHD (n = 14)	p‑value
Age at procedure (days)	314 (IQR: 175-332)	10 (IQR: 2-122)	<0.001
Age at procedure (approx. months)	10.5 (IQR: 5.8-11.1)	0.3 (IQR: 0.1-4.1)	-
Weight at procedure (kg)	5.9 (IQR: 4.5-6.0)	3.0 (IQR: 2.6-3.9)	<0.001
Procedural success	14/14 (100%)	13/14 (92.9%)	-
Any complication	2/14 (14.3%)	4/14 (28.6%)	0.65
30‑day survival	14/14 (100%)	7/14 (50.0%)	0.006

## Discussion

Transcatheter interventions have become indispensable in the management of critical CHD, particularly in resource-limited settings where surgical infrastructure is sparse and neonatal transport is precarious. Our single-centre series of 28 infants demonstrated an overall procedural success rate of 96% (27/28), an all-cause complication rate of 21.4% (6/28), and a 30-day survival of 75% (21/28), outcomes that vary substantially across physiological subgroups and are highly contextualised by patient complexity, underlying diagnosis, and the availability of staged surgical follow-up.

Patient selection and its influence on outcomes

The marked survival gradient across our three groups (100% (acyanotic), 100% (obstructive), 50% (cyanotic)) is not primarily a reflection of operator or institutional capability but of patient selection and case-mix complexity. Acyanotic and obstructive CHD cases presented at older ages (mean of 325 and 140 days, respectively), with stable haemodynamics, adequate weight (mean = 5.9 and 5.0 kg), and definitive rather than palliative intent. In contrast, cyanotic CHD cases presented at a median of 10 days, with a mean weight of 3.3 kg, in haemodynamic extremis, for palliative procedures that by design do not represent definitive repair. This selection effect must be explicitly acknowledged when interpreting between-group outcome comparisons.

For acyanotic CHD, device closures of PDA and VSD provide gentler alternatives to open surgery. In our cohort of nine cases, every procedure succeeded without major complications. While infants under 6 kg face heightened risks of arrhythmias and vascular injuries, meticulous patient management and refined techniques effectively curbed these threats [9-11]. Our results are consistent with contemporary series reporting success above 95% and minimal adverse events in infants over 5 kg with stable haemodynamics [12,13].

In obstructive CHD, BPV and coarctoplasty proved reliable. BPV, a staple since 1982, continues to demonstrate high efficacy and safety [14,15]. Coarctoplasty, despite risks like restenosis and aneurysms, serves as an essential interim step toward definitive surgery [16-19]; our series encountered just one deep vein thrombosis as a complication. Our excellent early outcomes with BPV and coarctoplasty (high success, one vascular complication, and no early deaths) mirror the long‑standing evidence base supporting these interventions as first‑line or staged strategies in obstructive lesions with low procedure‑related mortality [12,20].

Cyanotic CHD demanded more urgent measures. BAS remains a cornerstone for transposition of the great arteries (TGA) and kindred conditions, achieving 100% procedural success here, yet three infants succumbed within 30 days, highlighting the critical window for follow-up surgery. Delays from BAS to definitive repair beyond the first one to two weeks of life are associated with worse outcomes despite technically successful septostomy, which likely explains much of the mortality we observed, given the logistical challenges of referral and limited surgical access. Thus, our BAS‑related deaths are best interpreted as reflecting gaps along the care continuum (transport, ICU, and surgery) rather than intrinsic inadequacy of the procedure [13]. RVOT stenting offered a compelling alternative to systemic-pulmonary shunts in select TOF patients; however, post-procedural care and definitive surgery remain a major challenge due to resource-poor settings as compared to other studies [21-23]. PDA stenting, pioneered in 1992, is gaining favour over Blalock-Taussig-Thomas shunts for its reduced morbidity; however, our group saw a stark 57% 30-day mortality, driven primarily by sepsis and pneumonia. The substantially higher mortality after PDA stenting in our series is therefore best attributed to severe pre‑procedure instability, high sepsis burden, limited intensive care resources, and constrained or delayed access to subsequent staged repair, all common challenges highlighted in critical CHD cohorts from India and other low‑resource settings, rather than to the stent strategy per se [24-30].

Small subgroup sizes and statistical caution

The subgroup-level survival percentages, particularly 40% for BAS (2/5) and 43% for PDA stenting (3/7), must be interpreted with extreme caution given the small denominators. A single additional survivor in each subgroup would shift BAS survival to 60% and PDA stenting to 57%; one additional death would shift them to 20% and 29%, respectively. The 95% confidence intervals for these proportions are necessarily very wide: for BAS, 95% CI: 5.3-85.3%; for PDA stenting, 95% CI: 9.9-81.2%. These figures are therefore descriptive and hypothesis-generating, not inferential, and should not be used to draw conclusions about the relative efficacy of individual procedures. Larger prospective multicentre Indian registries, analogous to the CHRONIK neonatal registry from South India, are urgently needed to generate sufficiently powered subgroup estimates [31].

Limitations

This work underscores the feasibility and impact of transcatheter interventions at a tertiary centre in a tier-3 Indian city, effectively narrowing the paediatric cardiology care divide where surgical options falter. Yet, this study carries the inherent limitations of a retrospective single-centre design with a small sample (n = 28), which constrains statistical power, limits adjustment for confounders, and precludes generalisability to other Indian centres with different case-mixes or infrastructure. The absence of long-term follow-up data beyond 30 days prevents assessment of interstage mortality, pulmonary artery growth, reintervention rates, and eventual surgical outcomes, the most clinically meaningful endpoints for palliative procedures. Surgeon-dependent variability in patient selection and procedural technique cannot be quantified retrospectively. Missing data for some baseline variables (weight, ejection fraction) introduces potential ascertainment bias. Despite these limitations, this series adds to the small but growing body of real-world evidence from resource-limited Indian centres and provides procedure-specific outcome data that can inform future prospective registry design.

## Conclusions

Transcatheter interventions potentially can provide important first-line or bridging options for selected infants with CHD, especially when timely surgery or neonatal transport is not feasible. In resource-constrained settings, these procedures may facilitate haemodynamic stabilisation and create an opportunity for referral to centres with surgical capability, and in some anatomies, they can serve as definitive therapy. Incremental strengthening of transcatheter services, alongside improvements in peri-procedural intensive care and surgical access, has the potential to reduce unmet needs in paediatric cardiac care without replacing the essential role of surgery.
